# Double Recombinant Vaccinia Virus: A Candidate Drug against Human Glioblastoma

**DOI:** 10.3390/life11101084

**Published:** 2021-10-14

**Authors:** Natalia Vasileva, Alisa Ageenko, Maria Dmitrieva, Anna Nushtaeva, Sergey Mishinov, Galina Kochneva, Vladimir Richter, Elena Kuligina

**Affiliations:** 1 Institute of Chemical Biology and Fundamental Medicine, Siberian Branch of the Russian Academy of Sciences, Akad. Lavrentiev Ave. 8, 630090 Novosibirsk, Russia; a.ageenko@g.nsu.ru (A.A.); imaria819@gmail.com (M.D.); nushtaeva.anna@gmail.com (A.N.); richter@niboch.nsc.ru (V.R.); kuligina@niboch.nsc.ru (E.K.); 2 LLC “Oncostar”, R&D Department, Ingenernaya Street 23, 630090 Novosibirsk, Russia; 3 Novosibirsk Research Institute of Traumatology and Orthopedics n.a. Ya.L. Tsivyan, Department of Neurosurgery, Frunze Street 17, 630091 Novosibirsk, Russia; smishinov@yandex.ru; 4 The State Research Center of Virology and Biotechnology “VECTOR”, Department of Molecular Virology of Flaviviruses and Viral Hepatitis, Novosibirsk Region, 630559 Koltsovo, Russia; kochneva@vector.nsc.ru

**Keywords:** glioblastoma, oncolytic virus, VV-GMCSF-Lact, glioma stem cells, patient-derived glioblastoma cultures

## Abstract

Glioblastoma is one of the most aggressive brain tumors. Given the poor prognosis of this disease, novel methods for glioblastoma treatment are needed. Virotherapy is one of the most actively developed approaches for cancer therapy today. VV-GMCSF-Lact is a recombinant vaccinia virus with deletions of the viral thymidine kinase and growth factor genes and insertions of the granulocyte–macrophage colony-stimulating factor and oncotoxic protein lactaptin genes. The virus has high cytotoxic activity against human cancer cells of various histogenesis and antitumor efficacy against breast cancer. In this work, we show VV-GMCSF-Lact to be a promising therapeutic agent for glioblastoma treatment. VV-GMCSF-Lact effectively decreases the viability of glioblastoma cells of both immortalized and patient-derived cultures in vitro, crosses the blood–brain barrier, selectively replicates into orthotopically transplanted human glioblastoma when intravenously injected, and inhibits glioblastoma xenograft and metastasis growth when injected intratumorally.

## 1. Introduction

Glioblastoma is the most frequent and lethal primary brain malignancy with a median patient survival of about 15 months [1]. The standard treatment for this disease includes maximal surgical resection followed by radio- and/or chemotherapy [2]. Regrettably, however, no significant advances in the treatment of glioblastoma have been achieved in recent decades. Accordingly, the search and development of new, more effective methods and drugs for the treatment of glioblastoma is a very urgent task today.

One of the promising approaches to the treatment of malignant neoplasms, including glioblastoma, is therapy by oncolytic viruses (OVs). The distinctiveness of virotherapy lies in the dual mechanism of action, which is based on the tumor-selective oncolysis and the induction of systemic and adaptive immune responses of the organism [3]. Several drugs based on OVs are at various stages of clinical trials as anticancer agents for glioblastoma treatment. One of them, DNX-2401 (Delta-24-RGD), is a recombinant adenovirus with a deletion in the *e1a* gene and an insertion of the sequence encoding the RGD motif into the fibrillar protein gene. In phase I clinical trials, this drug had no dose-related toxic effects. In a phase Ib randomized trial of DNX-2401 versus DNX-2401 plus interferon gamma (IFN-γ) for recurrent glioblastoma, the overall survival rate at 1 and 1.5 years for all enrolled patients was 33% and 22%, respectively; thus, the use of combinatorial therapy (DNX-2401 + IFN-γ) did not improve patient survival [4]. The combination of DNX-2401 (intratumoral) with anti-PD-1 antibodies (pembrolizumab) (intravenous) is currently being evaluated in a phase II study for recurrent glioblastoma (NCT02798406). Another OV undergoing trials is also promising. A genetically modified third-generation herpes simplex virus, G47Δ targeting IL-12, was evaluated in a phase II clinical study in GBM patients who received repeated intratumoral stereotaxic injections in addition to temozolomide. The statistical significance was above the criteria for early termination; therefore, this trial was terminated early. Since G47∆ has been recognized by the Japanese government as a revolutionary therapeutic drug, an accelerated approval is expected [5]. Based on clinical trial data, a combination of different approaches, including virotherapy, could lead to a significant breakthrough in the glioblastoma treatment. Despite the promising clinical trials described, no oncolytic virus has yet been approved for the treatment of this malignancy [6]. This is associated with many existing problems: the presence of natural barriers such as the blood–brain barrier (BBB); the emergence of resistance pathways; intra- and interheterogeneity of glioblastoma, particularly the occurrence of glioma stem cells (GSCs); and the suboptimal pre-clinical and clinical trial designs [7]. Therefore, when developing and testing a novel drug against glioblastoma, these and other features must be taken into account.

In collaboration with the State Research Center of Virology and Biotechnology “Vector” on the basis of the Russian L-IVP strain of the vaccinia virus, we have created a recombinant virus VV-GMCSF-Lact, which contains deletions of viral thymidine kinase (*tk*) and growth factor (*vgf*) gene fragments, in the regions of which the genes of human granulocyte–macrophage colony-stimulating factor (GM-CSF) and the oncotoxic protein lactaptin are inserted, respectively. Developed in our laboratory, lactaptin is a fragment of human milk kappa-casein (residues 57–134) that induces the apoptotic death of various cultured cancer cells [8]. The combination of the *tk* and *vgf* gene deletions provides the virus with additional targeting of tumor cells and dramatically reduces its virulence against healthy cells. The expression of transgenes, GM-CSF and lactaptin, stimulates the antitumor immune response and induces apoptotic death of tumor cells, respectively [9]. The cytotoxic VV-GMCSF-Lact activity against human cancer cells of various histogenesis was shown, as was the antitumor efficacy in human breast cancer. Moreover, preclinical studies of a VV-GMCSF-Lact-based anticancer drug for the treatment of human breast tumors have been successfully completed. The drug is recommended for clinical trials.

The subject to be addressed in this present study was to investigate the therapeutic efficacy of VV-GMCSF-Lact against human glioblastoma. Using various in vitro models, we have shown that VV-GMCSF-Lact is able to suppress the growth of glioblastoma cells in both immortalized and patient-derived cell cultures. Moreover, the results of evaluating the abundance of cells carrying GSC markers suggest cultures with a large number of CD133+/CD44+ cells are more sensitive to the virus’ cytotoxic effects. As a second step, the VV-GMCSF-Lact biodistribution in SCID mice with orthotopically transplanted U87 MG tumors was examined. We have demonstrated that VV-GMCSF-Lact is not only able to cross the BBB when intravenously injected but can also replicate selectively in cancer cells. Finally, VV-GMCSF-Lact’s antitumor efficacy against cell-line-derived and patient-derived xenografts has been shown.

## 2. Materials and Methods

### 2.1. Cell Lines

Human U87 MG and U343 MG cell lines were obtained from the Russian cell culture collection (Russian Branch of the ETCS, St. Petersburg, Russia). The cells were cultivated in Minimum Essential Medium α (MEM α; Sigma-Aldrich, MS, USA) with 10% FBS (Gibco BRL Co., Gaithersburg, MD, USA), 2 mM L-glutamine (Sigma-Aldrich, MS, USA), 250 mg/mL amphotericin B, and 100 U/mL penicillin/streptomycin (Gibco BRL Co., Gaithersburg, MD, USA) at 37 °C in a humidified atmosphere containing 5% CO_2_.

Cancer tissue samples (MG1, MG4, BR1.20, and BR3.20) were obtained with informed consent from patients at the Novosibirsk Research Institute of Traumatology and Orthopedics n.a. Ya.L. Tsivyan (Novosibirsk, Russia). Tissue specimens were mechanically dissociated in Iscove’s modified Dulbecco’s media (IMDM, Sigma-Aldrich, MS, USA). Specimens dissociated into single cells were washed with 10× excess of phosphate-buffered saline (PBS), and separated cells were collected by centrifugation at 300× *g*. Cells were plated in IMDM medium with 10% FBS, 2 mM L-glutamine, 100 U/mL penicillin, 100 μg/mL streptomycin, and 250 mg/mL amphotericin B for cell adhesion. In the next steps, cells were cultured in complete IMDM medium supplemented with Mito + Serum Extender (BD Biosciences–Discovery Labware, San Jose, CA, USA), 2 mM L-glutamine, 100 U/mL penicillin, 100 μg/mL streptomycin, and 250 mg/mL amphotericin B, and were cultivated in 6-well plates at 37 °C in a humidified atmosphere containing 5% CO_2_. When 70–80% confluence was reached, cells were harvested using Triple-Express (GIBCO, Thermo Fisher, Waltham, NY, USA) and sub-cultured for further experiments. For neurosphere formation, MG1 and MG4 cells were cultured in Dulbecco’s Modified Eagle Medium: Nutrient Mixture F-12 (DMEM:F12, Sigma-Aldrich, MS, USA) supplemented with B-27 and N-2 supplements, human FGF-basic recombinant protein (Gibco BRL Co., Gaithersburg, MD, USA), and human EGF recombinant protein (Sigma-Aldrich, MS, USA) in non-treated cell culture dishes (Eppendorf, Hamburg, Germany) at 37 °C in a humidified atmosphere containing 5% CO_2_.

### 2.2. Virus

Recombinant vaccinia virus VV-GMCSF-Lact was kindly provided by Kochneva G.V. (State Research Center of Virology and Biotechnology “Vector”, Novosibirsk Region, Russia). Recombinant VV-GMCSF-Lact was engineered from Lister strain (L-IVP) vaccinia virus and has deletions of the viral thymidine kinase *(tk)* and vaccinia growth factor *(vgf)* gene fragments in the regions where the genes of human GM-CSF and the oncotoxic protein lactaptin are inserted, respectively [9].

### 2.3. Animals

Female SCID mice (6–8 weeks old) were obtained from the SPF, Vivarium of the Institute of Cytology and Genetics SB RAS (Novosibirsk, Russia). Mice were kept in the same room within a specific pathogen-free animal facility with a regular 12/12 h light/dark cycle at a constant room temperature of 22  ± 2 °C and a relative humidity of approximately 45 ± 15%.

### 2.4. Cell Viability Assay

Cell viability was detected using the Deep Blue Cell Viability Kit (BioLegend, San Diego, CA, USA). The cells that had reached 60% confluence in a 96-well plate were incubated for 72 h with VV-GMCSF-Lact; the multiplicity of infection ranged from 0.0012 to 10 PFU per cell. After incubation, commercial reagent was added into wells, and the optical density was measured according to the manufacturer’s instructions. Cell viability was determined relative to the viability of the control cells’ (100%) ± standard deviation in three independent experiments.

### 2.5. Flow Cytometry

Cells growing in 6-well plates were collected and incubated with phycoerythrin (PE)-conjugated mouse anti-human CD133 (Miltenyi Biotec, Bergisch Gladbach, Germany) and fluorescein isothiocyanate (FITC)-conjugated rat anti-human CD44 (R&D Systems, Minneapolis, MN, USA) antibodies in PBS supplemented with 0.5% fetal bovine serum and 2 mM EDTA for 30 min in ice. The analyses were performed using a FACSCantoII flow cytometer (BD Biosciences, Franklin Lakes, NJ, USA), and the data were analyzed by FACSDiva Software (BD Biosciences, Franklin Lakes, NJ, USA).

### 2.6. VV-GMCSF-Lact Biodistribution in SCID Mice with Orthotopically Transplanted U87 MG Tumors and in Healthy SCID Mice

U87 MG cells (5 × 10^5^ cells) were orthotopically transplanted into five SCID mice. When, according to MRI scanning, the tumors reached a size of 4 mm^3^, the mice were treated with a VV-GMCSF-Lact intravenous injection with a dose of 5 × 10^7^ PFU/mice twice, with an interval of 7 days. At 7 days after the last administration, all mice were euthanized and blood, tumor, brain, lung, kidney, and liver samples for further DNA extraction were taken. VV-GMCSF-Lact quantity was estimated by a qPCR assay.

To estimate VV-GMCSF-Lact biodistribution in healthy mice, three SCID mice without transplanted tumors were treated with VV-GMCSF-Lact intravenous injection with a dose of 5 × 10^7^ PFU/mice twice, at an interval of 7 days. At 7 days after the last administration, all mice were euthanized and blood, tumor, brain, lung, kidney, and liver samples for further DNA extraction were taken. VV-GMCSF-Lact quantity was estimated by a qPCR assay.

Determination of the recombinant vaccinia virus VV-GMCSF-Lact content in the blood and organs of mice was based on DNA extraction from samples of the test material and simultaneous amplification of DNA regions of the recombinant vaccinia virus and DNA of the mouse *rpl30* gene. The DNA of the mouse *rpl30* gene was used as an endogenous internal control. To carry out the amplification reaction in the multiplex format, two sets of primers were used: Lact-FAM primers targeting the lactaptin gene and the virus flank region (the region of the virus genome flanking the inserted lactaptin gene from the 3’ end), and Rpl30-ROX, targeting the *rpl30* gene fragment mouse. The following specific primers were used:

Lact-FAM:

Forward 5′-TACCATCGGCGGATCACATC-3′;
Reversed 5′-AGCACAATACCGGGAGATGG-3′;
Probe  5′-FAM-TCAGAAAACCCAAACACTACAACGTCA-BHQ-1-3′;

Rpl30-ROX:

Forward 5′-GATCAGACAAGGCAAAGCGAAG-3′;
Reversed 5′-GACCCCAGTTTTAGCCAACATG-3′;
Probe  5′-ROX-CTGTCCAGCTTTGAGGAAATC–BHQ2-3′.

DNA extraction from blood and tissue species was performed using isolation of genomic DNA from cells and tissues and a blood kit (Biolabmix, Novosibirsk, Russia) according to the manufacturer’s instructions. PCR reaction was performed with BioMaster HS-qPCR (Biolabmix, Novosibirsk, Russia). For quantitative PCR analysis, standard samples were prepared with a known concentration of target DNA. The detection of a fluorescent signal was carried out directly during PCR using an amplifier with a system for detecting a fluorescent signal in "real time" CFX96 (BioRad, Hercules, CA, USA). The amount of virus in the samples was determined using an automatically constructed standard curve and BioRad CFX Manager software (Bio-Rad, Hercules, CA, USA).

### 2.7. VV-GMCSF-Lact Antitumor Efficacy against Subcutaneously Transplanted Human Glioblastoma Cells into SCID Mice

U343 MG, U87 MG cells (3 × 10^6^ cells per mouse, in PBS) and MG1ns, MG4ns (5 × 10^6^ cells per mouse, in PBS) were mixed with Matrigel (BD Bioscience, Franklin Lakes, NJ, USA) at a ratio of 1:1 and subcutaneously transplanted into SCID mice. The tumor volumes (V_t_) were measured and calculated using the following Equation (1):

V_t_ = (a^2^ × b)/2 (1)

where a is the shorter tumor diameter in mm, and b is the longer tumor diameter in mm.

When the tumor reached 100–120 mm^3^ in size, U87 MG, U343 MG, and MG4ns tumor-bearing mice were treated with VV-GMCSF-Lact intratumoral injections. The virus was administered intratumorally three or four times at an interval of 7 days with a dose of 10^7^ PFU/mouse. The control group received saline according to the same scheme.

To evaluate antimetastatic VV-GMCSF-Lact efficacy, U343 MG cells (3 × 10^6^ cells per mouse, in PBS) were subcutaneously transplanted on different sides of the SCID mice body for “tumor” and “metastasis” formation. The virus was injected only into the “tumor” according to the scheme described above.

The tumor growth inhibition (TGI) in all in vivo experiments was calculated using the following Equation (2):

TGI (%) = 1 − (V_t_/V_c_) × 100 (%) (2)

where V_t_ is the mean tumor volume of treated mice, and V_c_ is the mean tumor volume of control animals.

### 2.8. Ethical Statement

All the animal experiments were carried out in compliance with the protocols and recommendations for the proper use and care of laboratory animals (EEC Directive 86/609/EEC). The protocols were approved by the Committee on the Ethics of Animal Experiments of the Administration of the Siberian Branch of the Russian Academy of Science (protocol number: 68; date: 1 December 2020). The mice were housed under specific-pathogen-free (SPF) conditions in ventilated animal cabinets under controlled lighting conditions at 45 ±  15% humidity, 22 ± °C, with a 12/12 h light–dark cycle, and were allowed food and water ad libitum. The animals were euthanized by exposure to CO_2_.

### 2.9. Statistics

Significance was determined using a two-tailed Student’s *t*-test. All error bars represent standard deviation of the mean. *p* < 0.05 was considered to be statistically significant.

## 3. Results

### 3.1. VV-GMCSF-Lact Cytotoxic Activity against Human Glioblastoma Cells

To evaluate the VV-GMCSF-Lact cytotoxic activity in vitro, various human glioblastoma cells were used. U87 MG and U343 MG cells were taken as established glioblastoma models. MG1, MG4, BR1.20, and BR3.20 cells were obtained from patient tumor samples (patient-derived cell cultures). MG1 and MG4 cells were grown under two different conditions: a standard serum-containing medium and neurosphere-forming conditions (MG1ns and MG4ns cells). According to the literature, the cultivation of patient-derived cell cultures in a medium without serum and with the addition of growth factors and nutritional supplements allows for the maximization of the preservation of the phenotype and genotype of the primary tumor [10]. Thus, these cultures were subjected to various conditions to assess if these influenced the sensitivity to the VV-GMCSF-Lact action.

**Figure 1:**
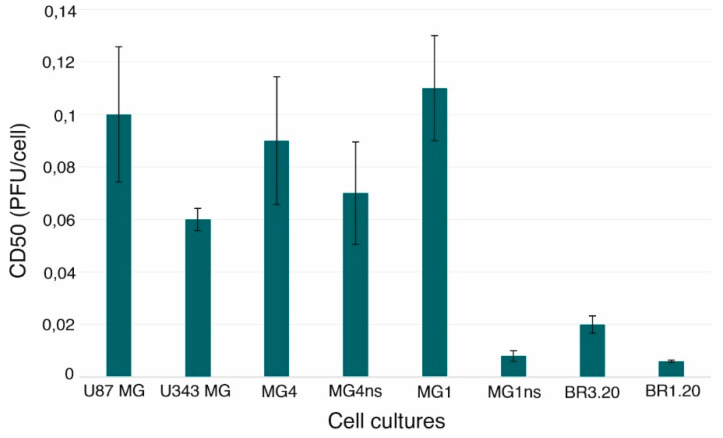
VV-GMCSF-Lact cytotoxic activity against human glioblastoma cells in vitro. Cells were infected with the recombinant virus (0.0012–10.0 PFU/cell) and incubated for 72 h. The 50% cytotoxic dose (CD50) was evaluated for each cell line using the Deep Blue Cell Viability kit. Statistical analysis included the results of three independent experiments. Significant differences between groups are presented in Table A1.

VV-GMCSF-Lact has been shown to have high cytotoxic activity against both immortalized and patient-derived human glioblastoma cell cultures (Figure 1).

Thus, cells of various cultures were differently sensitive to the virus action. U87 MG, MG1, and MG4 glioblastoma cells turned out to be more resistant to the VV-GMCSF-Lact action when compared to cells of other studied cultures. It is worth noting that cultivated under standard conditions, MG1 cells were more resistant to the virus oncolytic action than MG1ns. Additionally, there was a tendency of an increase in the MG4 sensitivity to the VV-GMCSF-Lact when grown under neurosphere-forming conditions.

### 3.2. CD133 and CD44 Expression in Cells with Different VV-GMCSF-Lact Sensitivity

**Figure 2:**
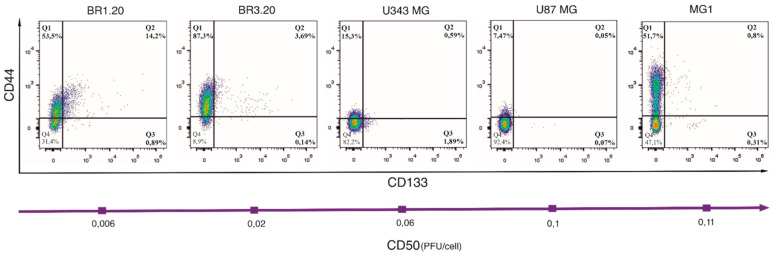
CD133 and CD44 expression profiles and their co-expression in MG1, BR1.20, BR3.20, U343 MG, and U87 MG cell cultures. Cell suspensions were labeled with PE-conjugated anti-CD133 and APC-conjugated anti-CD44 antibodies and analyzed by flow cytometry. The top portion (Q1, Q2) is CD44-positive; the right portion (Q2, Q3) is CD133-positive cells. The double-positive cells in the right-top quarter (Q2) are numbered as a percentage of the total cells. The purple arrow defines a decrease in the sensitivity of the cells of the studied cultures to VV-GMCSF-Lact.

Cancer stem cells are believed to be the most resistant tumor cell population to current therapy [11]. To evaluate whether there is an interdependence between the amount of glioma stem cells and the sensitivity of the studied cultures to the virus, we assessed the CD133 and CD44 levels, the most common markers of glioblastoma stem cells [12], in U87 MG, U343 MG, MG1, BR1.20, and BR3.20 cell cultures by flow cytometry (Figure 2).

CD44 and CD133 expression was lower or not detected in MG1, U87 MG, and U343 MG cultures compared to BR1.20 and BR3.20. Herewith, BR1.20 was characterized by a large number of CD133-positive cells (~15%) and BR3.20 by a large number of CD44-positive cells (~90%). It should be noted that the co-expression of CD133 and CD44, as well as the expression of CD133 alone, was observed at a higher level in cultures that were more sensitive to the VV-GMCSF-Lact cytotoxic action.

### 3.3. VV-GMCSF-Lact Ability to Cross the Blood–Brain Barrier

**Figure 3:**
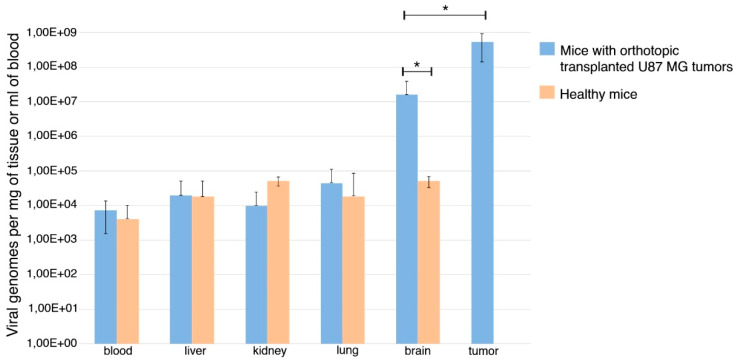
VV-GMCSF-Lact biodistribution in SCID mice with orthotopic transplanted U87 MG tumors and in healthy SCID mice. Tissues were collected from mice administered with VV-GMCSF-Lact. The amount of the virus was determined by a real-time PCR assay using standard VV-GMCSF-Lact dilutions. The data represent the mean ± SD of five mice for a group of animals with transplanted tumors and the mean ± SD of three mice for healthy animals. The asterisks indicate a significant difference between groups (* *p* < 0.05).

To understand whether VV-GMCSF-Lact is able to cross the BBB, we investigated the virus biodistribution in the organs and tissues of SCID mice with orthotopic transplanted U87 MG tumors. VV-GMCSF-Lact was administered intravenously twice, with a dose of 5 × 10^7^ PFU/mice at an interval of 7 days. At 7 days after the last injection, mice were euthanized and blood, tumors, and organs (liver, lung, kidney, and brain) were taken for further analysis by a real-time PCR assay. The data obtained showed VV-GMCSF-Lact to be distributed throughout all body tissues, including the brain. Moreover, the amount of virus in the tumor was significantly higher than in other organs and tissues (Figure 3).

It should also be mentioned that the amount of VV-GMCSF-Lact in the normal brain tissue was essentially more than in other organs. This may be due to infiltration of the surrounding space by cancer cells with the subsequent virus replication. Nevertheless, a significant distinction between the amount of virus in the tumor and the normal brain was identified.

### 3.4. VV-GMCSF-Lact Antitumor and Antimetastatic Efficacy against Human Glioblastoma

To estimate VV-GMCSF-Lact’s antitumor efficacy, a subcutaneous xenograft model was used. U87 MG and U343 MG tumors, subcutaneously transplanted into SCID mice, were utilized as established models.

**Figure 4:**
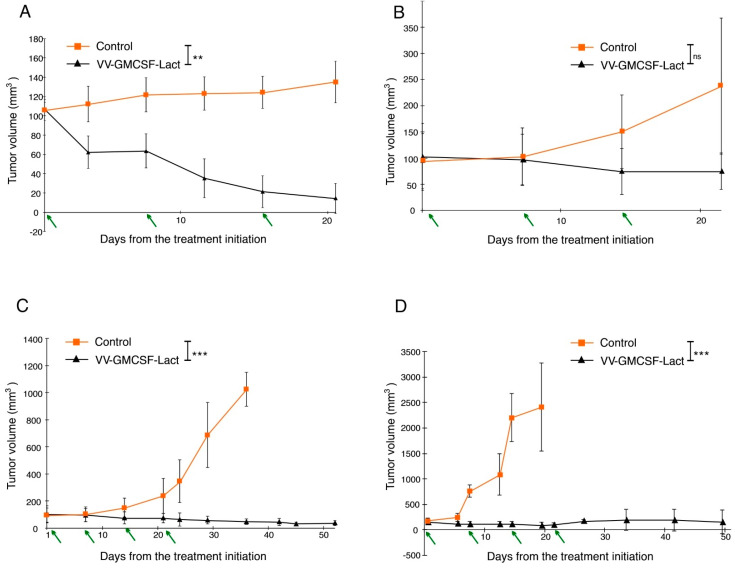
VV-GMCSF-Lact antitumor efficacy against human glioblastoma in vivo. The data represent the mean ± SD of six mice. Arrows indicate the days of the drug injections. (**A**) U343 MG subcutaneous tumor volumes were decreased after i.t. treatment with VV-GMCSF-Lact. ** *p* < 0.01. (**B**) No effective inhibition of U87 MG subcutaneous tumor growth was achieved 21 days after treatment. ns: non-significant. (**C**) Growth of U87 MG subcutaneous tumors was inhibited after the fourth i.t. VV-GMCSF-Lact injection. *** *p* < 0.001. (**D**) MG4ns subcutaneous tumor volumes were decreased after i.t. treatment with VV-GMCSF-Lact. *** *p* < 0.001.

U87 MG and U343 MG tumor-bearing mice were treated with VV-GMCSF-Lact intratumoral injections. The tumor volume at the treatment start was about 100–120 mm^3^. The virus was administrated three or four times at a dose of 10^7^ PFU/mice at an interval of 7 days. The animals of the control group were treated with saline according to the same scheme. Inhibition of tumor growth was determined by comparing the tumor volumes of the experimental and control group animals. Twenty-one days after the first virus injection (or six days after the third injection), U343 MG tumor growth inhibition by VV-GMCSF-Lact was 84%, and about 30% of tumors were completely eliminated (Figure 4A). In the treatment of the mice with the U87 MG tumor, the suppression of tumor growth 22 days after the first injection was 68% (Figure 4B). Since no effective inhibition of tumor growth was achieved, a fourth VV-GMCSF-Lact injection was given. When the mice with U87 MG tumors were in the experiment, within 53 days (Figure 4C) the tumor growth inhibition by VV-GMCSF-Lact was 96%. It is worth mentioning here that there were no completely healed tumors. The animals of the control group were euthanized at day 37 of the experiment due to the development of necrosis foci in the tumors.

Accordingly, the obtained cultures MG1ns and MG4ns were investigated for the ability to form tumors during subcutaneous transplantation into SCID mice. When transplanting 5 × 10^6^ cells of both MG1ns and MG4ns into mice, after 3 weeks, on average, the formation of tumor nodes was observed. However, the growth of MG4ns tumors proceeded more efficiently. Thereby, MG4ns xenografts were taken as a more relevant glioblastoma model with which to assess VV-GMCSF-Lact antitumor efficacy. VV-GMCSF-Lact has been shown to effectively inhibit MG4ns tumor growth. Twenty-two days after the first virus injection, the tumor growth inhibition was 97%. Only 10% of the tumors were completely eliminated by the 58th day of the experiment (Figure 4D). Animals of the control group were euthanized on the 22nd day of the experiment because the tumor volumes were about 2000 mm^3^ and exceeded the values allowed for further work with animals.

**Figure 5:**
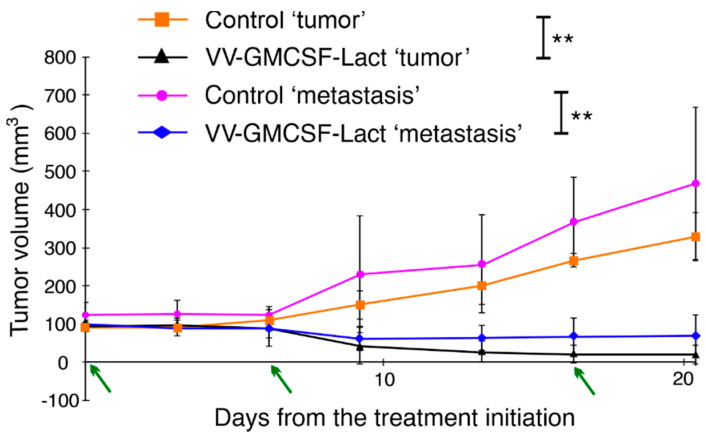
Dynamics of U343 MG “tumor” and “metastasis” growth during VV-GMCSF-Lact treatment. Growth of U343 MG subcutaneous “tumor” and “metastasis” was inhibited after i.t. treatment with VV-GMCSF-Lact. ** *p* < 0.01. The data represent the mean ± SD of six mice. Arrows indicate the days of the drug injections.

Previously, our colleagues showed that a recombinant vaccinia virus with deletions of the *tk* and *vgf* genes and with the insertion of the green fluorescent protein gene (GFP2) VVdGF-GFP2 was able to reach metastasis [13]. The recombinant virus was intratumorally injected into nude mice with subcutaneously transplanted human carcinoma A431 cells at points distant from each other. According to the UV images of mice, VVdGF-GFP2 was registered as in metastases after 2 days. To test whether VV-GMCSF-Lact was capable of not only reaching but also suppressing glioblastoma metastases, we used a model of metastasis: the subcutaneous transplantation of U343 MG cells on different sides of the SCID mice body for “tumor” and “metastasis” formation. The tumor volume at the treatment start was about 100–120 mm^3^. Animals were treated for the “tumor” with VV-GMCSF-Lact at a dose of 10^7^ PFU/mice or saline (three injections every 7 days). The “tumor” and “metastasis” volumes were measured. On day 7 after the last injection, we observed statistically significant (*p* < 0.05) suppression of both “tumor” and “metastasis” growth of VV-GMCSF-Lact-treated mice (Figure 5). Tumor growth inhibition was 93.9% for the “tumor” and 85.2% for “metastasis”.

## 4. Discussion

Oncolytic viruses are a promising approach for glioblastoma therapy, working by selectively inhibiting and killing cancer cells [14].

The vaccinia virus is the most utilized for therapeutic purposes—this virus is well-studied as it was used for the vaccination against smallpox and created a well-established safety record. However, since the native vaccinia virus can replicate not only in tumor cells, but also in healthy cells of the organism [15], for the development of antitumor drugs recombinants with deleted virulence genes are used. It has been shown that a genetically modified vaccinia virus suggests greater antitumor potency against human disease and reduced virus side effects caused by off-target replication. Lister strain virus with thymidine kinase gene deletion demonstrated superior antitumor potency and cancer-selective replication in vitro and in vivo, especially in human cancer cell lines and immune-competent hosts. This recombinant may be a particularly promising vaccinia virus strain for the development of the next generation of tumor-targeted oncolytic therapeutics [16].

We created a double recombinant vaccinia virus, VV-GMCSF-Lact, using the Lister strain as a parent. VV-GMCSF-Lact has deletions of the viral thymidine kinase and vaccinia growth factor genes in the regions where the genes of human granulocyte–macrophage colony-stimulating factor (GM-CSF) and the oncotoxic protein lactaptin are inserted. Transgenes GM-CSF and lactaptin were used to increase the antitumor efficacy of the recombinant strain [9]. GM-CSF stimulates antitumor immunity and lactaptin induces apoptosis of tumor cells. Proapoptotic protein lactaptin was also developed and investigated by the same authors’ team [17].

In our previous studies we compared the oncotoxicity of VV-GMCSF-Lact and VV-GMCSF-dGF (virus strain without lactaptin) to human tumor cells, including epithelial glioblastoma U87MG, and found that VV-GMCSF-Lact induced the death of all cultured cancer cells more efficiently than recombinant VACV coding only GM-CSF. We also estimated tumor growth inhibition and survival outcomes after VV-GMCSF-Lact and VV-GMCSF-dGF treatment using immunodeficient (MDA-MB-231 human breast cancer) and immunocompetent (RLS lymphosarcoma) mice models. Additionally, we also observed intravenous and intratumoral injections of VV-GMCSF-Lact to be more effective than VV-GMCSF-dGF treatment [9]. Thus, lactaptin expression increased the toxicity of recombinant virus to cancer cells and its antitumor efficacy in vivo.

In this study, based on our previously obtained results, we showed that the recombinant vaccinia virus VV-GMCSF-Lact could be a candidate drug for glioblastoma treatment. Challenges exist regarding the clinical application of OVs, such as the therapeutic resistance of glioblastoma, immunosuppressive microenvironments, or the presence of the blood–brain barrier [18,19]. There also is complicated internal heterogeneity of glioblastoma at the cellular and molecular level [20] as well as with the selection of relevant models for preclinical research [10]. One of the common preclinical models for studying the biology of glioblastoma and its response to therapy are cell cultures obtained from patient tumor samples and cultured under conditions of neurosphere formation [21]. Growth conditions in the absence of serum, but with the addition of growth factors and nutritional supplements, allow the maximum preservation of both the phenotype and genotype of the primary tumor, as well as the molecular and phenotypic characteristics of tumor stem cells. In our study, when cultured under standard conditions with serum, the patient-derived cell cultures turned out to be more resistant to the oncolytic action of the virus than cells grown in the form of neurospheres. It can be assumed that cancer cells cultured with the addition of epidermal growth factor (EGF), basic fibroblast growth factor (bFGF), and nutritional supplements acquire certain properties, due to which the treatment with VV-GMCSF-Lact is more effective. Amplification of the epidermal growth factor receptor (EGFR) gene is known to be observed in more than 50% of glioblastoma cases [22]. The addition of EGF in the medium is able to stimulate the EGFR-dependent signaling pathways, such as phosphoinositide 3-kinase (PI3K)/protein kinase B (AKT) [23,24]. Subsequently, phosphorylated p21-activated kinase 1 (PAK1) participates in the cytoskeleton transformation and microtubule dynamics process, and this mediates the blabbing of the cell membrane, the severity of which directly affects the number of viral particles entering the host cell [25,26]. Differences in the activation level of such signaling pathways are also one of the possible reasons to explain the distinct responses of glioma cells to the VV-GMCSF-Lact action. We also assumed that the higher sensitivity of the glioblastoma cells to the VV-GMCSF-Lact action was due to the high number of cells carrying CD133, also known as prominin-1, glioma stem cell markers. Recent studies have shown that this marker plays an important role in various types of cancer [27,28]. It was shown that phosphorylation of tyrosine-828 and tyrosine-852 in the CD133 cytoplasmic domain can regulate the CD133 interaction with the SH2-domain-containing proteins, which are able to affect intracellular signaling pathways, for example, to mediate the activation of the PI3K/AKT pathway [29]. There is also CD133-AKT-Wnt signaling axis in human glioblastoma, according to which CD133 acts as a cellular receptor mediating AKT-dependent activation of the Wnt pathway [30]. Therefore, upregulated molecular processes in CD133-positive glioblastoma cells can promote more efficient penetration of VV-GMCSF-Lact into cells and its active oncolytic action. An effective VV-GMCSF-Lact impact on GSCs demonstrates its promising application as a therapy for recurrent glioblastoma. Treating the resection cavity with the virus or intravenous virus administration after surgery can reduce the risk of relapse by inhibiting the growth of GSCs. It is known that a population of GSCs is capable of self-renewal, proliferation, and invasion, and can cause local recurrence around the surgical site and/or non-local recurrence in the remainder [31,32]. Possible combinatorial therapy is used to reduce the risk of relapse. According to clinical trials, in glioblastoma, oncolytic viruses are used in combination with chemotherapy (cyclophosphamide [33]), immune checkpoint inhibitors (pembrolizumab [34]), and other therapeutic approaches.

As mentioned above, apart from identifying the mechanisms of tumor sensitivity to the therapy, there are also other challenges in developing anticancer agents. One of them is the presence of the BBB. Although the BBB is often disrupted in glioblastoma, this does not necessary mean that antitumor agents, unhindered, penetrate the tumor tissue. First, some BBB sections can be intact [35], and, secondly, there is also the blood–brain–tumor barrier (BBTB). While the BBTB is characterized by permeability, it does not guarantee meaningful drug penetration and accumulation due to the glioblastoma specificity [36,37]. Delivery of drugs for the treatment of glioblastoma is carried out by such methods as systemic delivery (intravenous, intra-arterial), local delivery (intranasal delivery, solid-based implant delivery, intratumoral delivery, and convection-enhanced delivery) [38]. The most common method of OV delivery is intratumoral injection because of the reduced effect of the immune response and the resolution of the difficulties associated with the BBB and BBTB presence, in addition to safety reasons [19]. At the same time, it is worth noting that a flawless OV should be administered systemically, considering that this way is less invasive and allows one to overcome limitations arising from the virus solution backflow up the catheter or the needle [17,37]. We have shown that VV-GMCSF-Lact can penetrate the BBB when intravenously injected into SCID mice with orthotopically transplanted U87 MG tumors. That the virus is able to replicate in cancer cells thereby provides additional virus accumulation in the tumor tissue. However, the impact of circulating antibodies on the efficacy of the virus delivery should be considered. Neutralizing anti-viral antibodies against oncolytic viruses is believed to reduce the multiplicity of infection and limit the oncolytic virus potential for systemic delivery [7]. However, it is known that poxviruses have evolved many proteins with which to disrupt host immune signaling and to deceive the host immunity [39]. Nonetheless, the literature indicates that the activation of anti-viral antibodies occurs regardless of the virus administration route [40,41]. According to the results of numerous clinical trials of Pexa-Vec, created on the basis of the recombinant vaccinia virus JX-594, the antitumor effect of the drug persisted, despite the high level of antibodies against the virus. Moreover, even pre-existing neutralizing antibodies in patients vaccinated earlier did not abrogate the antitumor effect of Pexa-Vec [42,43]. However, to date, methods are actively being developed to overcome virus neutralization [44]. Accordingly, glioblastoma treatment with oncolytic viruses will be suitable not only if the virus has the ability to penetrate the BBB, which we have successfully demonstrated for VV-GMCSF-Lact, but also if a sufficient accumulation of the virus in the brain tumor tissue is observed when the immune response is exposed. Immunodeficient rodent models did not make it possible to determine the involvement of the immune response [45]. Thus, the VV-GMCSF-Lact antitumor effect, when treating immunocompetent animals, is to be examined.

However, immunodeficient mice models make it possible to evaluate the antitumor efficacy of therapeutic agents for treating tumors in preclinical trials using cell lines obtained from patients [46,47]. Xenografts obtained by transplanting cells of immortalized lines have the advantages of high engraftment and growth rates, good reproducibility, and reliable growth and disease progression [48]. However, these cells undergo differentiation during long-term cultivation, and thereby the cell-line-derived xenografts no longer reflect the cellular heterogeneity of a malignant tumor [10,49]. Moreover, the genotypes of glioblastoma cell-line-derived xenograft models also differ from those of the original patient tumors [50,51]. Thus, doubts arise as to whether such models reflect the true glioblastoma response to treatment and how appropriate it is to limit them only to preclinical studies of anticancer agents. In contrast, patient-derived glioblastoma cells cultivated under neurosphere-forming conditions continue to maintain histological and molecular genetic similarity with the primary tumor [10,52]. Thus, murine tumor models obtained from patient-derived cultures have been shown to mimic many aspects of the parental tumor. However, not all patient-derived human glioblastoma cell cultures are successfully cultured as neurospheres, and not all obtained cultures are capable of forming tumors when transplanted into immunodeficient animals [46]. We were able to demonstrate the antitumor efficacy of VV-GMCSF-Lact against xenografts of both immortalized cultures and those obtained from patient tumor samples. Moreover, we showed the antimetastatic efficacy of VV-GMCSF-Lact using a model of metastasis. Despite the fact that glioblastomas rarely metastasize, there might be cases when glioblastoma cells enter the extracranial space, and surgery is one of the causes of the extraneural spread of glioblastoma cells [53,54]. Therefore, this issue deserves attention when developing novel glioblastoma therapy approaches. Surgical resection, the first-line therapy for brain tumors, is followed by radiotherapy, which can facilitate vascular invasion and tumor dissemination since neovascularization is considered to be one of the causes for metastasogenesis [55]. In addition, glioblastoma is characterized by a strong invasive potential of both cancer stem cells and differentiated cells [56]. Spreading into the surrounding brain tissues, glioblastoma cells cause either tumor recurrence or metastases. The most common is white matter metastases [57]. Additionally, the dissemination of glioblastoma cells can pass through the cerebrospinal fluid and lymphatic drainage from [58], while the most common sites of metastases are the lungs and pleura, lymph nodes, and bones [59]. VV-GMCSF-Lact is able to locate the metastases and exhibits significant antimetastatic activity when the intratumoral virus is injected. The last finding is important since, today, the preferred method of oncolytic virus delivery to glioblastoma is nevertheless intratumoral administration.

It is necessary to highlight the importance of assessing the effect of the VV-GMCSF-Lact on glioblastoma in an immunocompetent animal model, as well as the mechanisms underlying the effectiveness of virotherapy with the VV-GMCSF-Lact. The data obtained in this work and those that will be obtained later will allow a more comprehensive study of the possibility of further use of the virus in the clinic, as well as the development of the best therapy strategies.

## 5. Conclusions

The data obtained allowed us to conclude that VV-GMCSF-Lact is a perspective candidate drug for glioblastoma treatment. First, VV-GMCSF-Lact is able to cross the BBB, and this is one of the essential criteria in developing agents for brain tumor therapy. The virus also has both high cytotoxic and antitumor efficacies against human glioblastoma and antimetastatic efficacy in pre-clinical models. In the future, it will be necessary to study the mechanisms that determine the sensitivity of personal glioblastoma tumors to the VV-GMCSF-Lact action. In addition, it will be necessary to assess the effect of the immune response on the virus therapy. The results will permit the development of a glioblastoma treatment strategy using VV-GMCS-Lact with the corresponding combination of drugs, and will increase the survival rate and improve the quality of patient life in cases of clinical trial success. However, it can already be concluded that VV-GMCSF-Lact is a very promising candidate. It can be used in combination with chemotherapy agents and with radiotherapy after surgical removal of the tumor to treat the resection cavity, as well as with different routes of administration.

## Data Availability

The study has not created publicly available archived datasets.

## Appendix A

**Table A1:** Statistical differences between the values of the glioblastoma cell sensitivity to the cytotoxic effect of VV-GMCSF-Lact. Comparison data of the sensitivity of various glioblastoma cell cultures with each other are presented (* *p* < 0.05 and ** *p* < 0.01; ns: non-significant; ×: no comparison).

Cell Culture	U87 MG	U343 MG	MG4	MG4ns	MG1	MG1ns	BR3.20	BR1.20
U87 MG	×	*	ns	ns	ns	**	**	**
U343 MG	*	×	ns	ns	*	**	**	**
MG4	ns	ns	×	ns	ns	**	**	**
MG4ns	ns	ns	ns	×	ns	**	**	**
MG1	ns	*	ns	ns	×	**	**	**
MG1ns	**	**	**	**	**	×	*	ns
BR3.20	**	**	**	**	**	*	×	*
BR1.20	**	**	**	**	**	ns	*	×
